# It takes two to tango: carers’ reflections on their participation and the participation of people with dementia in the James Lind Alliance process

**DOI:** 10.1186/s12877-020-01570-3

**Published:** 2020-05-14

**Authors:** Agnete Nygaard, Liv Halvorsrud, Asta Bye, Astrid Bergland

**Affiliations:** 1 Faculty of Health Sciences, OsloMet - Metropolitan University, 0130 Oslo, Norway; 2 Lørenskog municipality, the Centre for Development of Institutional and Home Care Services, Lørenskog, Akershus Norway; 3 Regional Advisory Unit in Palliative Care, Department of Oncology, Oslo University Hospital, Oslo, Norway

**Keywords:** Patient and public involvement, Dementia, Carers, James Lind Alliance, Thematic analysis

## Abstract

**Background:**

Worldwide, patient and public involvement (PPI) in health research has grown steadily in recent decades. The James Lind Alliance (JLA) is one approach to PPI that brings patients, carers and clinicians together to identify priorities for future research in a Priority Setting Partnership (PSP). Our study aim was to describe the reflections of informal carers of people with dementia on the possibility of participating in the JLA’s PSP process, for both themselves and the recipients of their care. In addition, we wanted to explore barriers to and facilitators of their participation.

**Methods:**

We conducted four focus groups with 36 carers of people with dementia**.** Thematic analysis was applied to analyse the data.

**Results:**

An overarching theme emerged from the participants’ reflections: “Creating empowering teams where all voices are heard”. The overarching theme incorporates the participants’ suggestions about the importance of equivalence in power, mutual agreement with and understanding of the goals, adequate support, openness about each partner’s tasks and the bonds needed between the partners to sustain the enterprise, and expectations of positive outcomes. From the overarching theme, two main themes emerged: “Interaction of human factors, the PSP process and the environment” and “The power of position and knowledge”. The overall results indicated that carers are willing to participate in PSP processes and that they thought it important for people with dementia to participate in PSP processes as well, even if some might need extra support to do so. The carers also identified the need for research topics that influence their everyday lives, policy development and healthcare services.

**Conclusions:**

Both carers and the people with dementia for whom they care are able to contribute to the PSP process when given sufficient support. The involvement of these groups is important for setting healthcare research agendas, developing research projects that increase awareness and knowledge about their circumstances and improving health professionals’, researchers’ and policymakers’ understanding of and insight into their unique situations.

## Background

There are nearly ten million new cases of dementia-related diseases worldwide every year [1]. As a significant global public health concern, dementia is currently a top priority among major research funding agencies [2]. Research shows that unpaid family and informal carers are major sources of support for people with dementia and are estimated to cover as much as 85–90% of total societal costs in high-income countries [3]. One study estimates that one in three people will care for a person with dementia during their lifetime [4]. Another notes that carers of those with dementia have a higher incidence of depression, anxiety, poor sleep quality and substance abuse or dependence [5].

Worldwide, patient and public involvement (PPI), defined as “experimenting with” – as opposed to “experimenting on” – patients or the public [6] in health research has grown steadily in recent decades [7]. Various policy directives promoting PPI have been introduced, and funding bodies increasingly require the integration of such involvement into research projects [8]. PPI allows active contribution through discussion and involvement in decision making about research design, acceptability, relevance, conduct, governance and dissemination [9]. However, users’ involvement in research suffers from a scattered and diverse evidence base in terms of utility and impact, as well as from weak conceptual and theoretical frameworks [10].

One approach that uses PPI is the James Lind Alliance (JLA), a non-profit initiative established in 2004 in the UK. The main focus of the JLA’s Priority Setting Partnerships (PSPs) is to unite patients and/or carers (or groups representing these) with clinicians (or groups representing them) in order to prioritize uncertainties related to research, also referred to as evidence uncertainties which is defined as no up-to-date, reliable systematic reviews of research evidence addressing the uncertainty exist. Up-to-date systematic reviews of research evidence show that uncertainty exists. According JLA is the term ‘treatment uncertainties’ changed to ‘evidence uncertainties’ which is about ‘treatment’, such as care, support and diagnosis [11]. The aim of the JLA method is to raise awareness among research funding groups about the issues that matter most to patients, carers and clinicians, and to ensure that clinical research is both relevant and beneficial to end users [12]. According to the JLA Guidebook [11], uncertainties and how they should be prioritized are core elements in the PSP process.

The PSP process facilitates and guides the identification and prioritization processes. The main steps described in the JLA Guidebook are (1) setting up a steering group to supervise all aspects of the study, (2) establishing a PSP, (3) assembling potential research questions, (4) processing, categorizing and summarizing the research questions and (5) determining the top 10 research priorities. The PSP process culminates in a priority-setting workshop based on the respondents’ rankings and consensus-based discussion [11].

In a quantitative review of JLA-led approaches to the PSP process, we found that the PSP process is useful for identifying research questions. None of the reviewed studies reported difficulties in developing their top 10 priorities but the participation of people with different health conditions depends on their having the capacity and resources to participate [13]. However, to our knowledge, only one study has examined the participants’ views on the PSP process and that study did so 2 years after their participation in the process [14]. Shepherd et al. [15] stated that research priority setting methods can assist researchers and policymakers to effectively target fields that has the greatest potential benefit. They had carried out a priority setting in long term care settings. Shepherd et al. used a modified Delphi technique to identify research topics and to develop consensus among health-care professionals in care home. Some common priorities in the study included a focus on the needs of cognitively impairment residents. The authors suggested that future research agenda should have focus on this group. Furthermore, they stated that further work is required to identify the priorities of other key stakeholders [15].

Given the uniqueness of living with dementia and mainstream researchers’ lack of experience-based knowledge, the inclusion of the “lived experience” of service users and consultants may contribute a critical real-world perspective to dementia research. To enhance understanding of the ways in which research can support both those with dementia and their carers, it is crucial to explore the perspectives of key stakeholders such as carers. As research programmes and funding agencies increasingly address priorities identified through processes such as that used by the JLA, it is important to understand stakeholders’ views of these processes. Therefore, our study aim was to describe the reflections of carers of people with dementia on how they and those for whom they care can be enabled to participate in the PSP process. In addition, we wanted to explore the barriers to and facilitators of their participation. In this way, the study might add important knowledge about ways to facilitate the participation of carers’ and people with dementia in the PSP process.

## Methods

### Design

We used an exploratory, qualitative design, following the guidelines of the Consolidated Criteria for Reporting Qualitative Studies (COREQ) [16] to describe carers’ reflections on their own potential participation and that of people with dementia in the PSP process. A semi-structured interview guide was created and used to guide focus group with carers of people with dementia (see Supplementary File).

### Recruitment and participants

Participants were recruited from the Norwegian Health Association’s yearly meeting at a hotel in Norway; all those asked by email to participate agreed to do so. The authors had not met the participants before the focus group. The participants in this purposive sample were former carers who had already joined or were in the process of joining the voluntary Peer Support Services run by the Norwegian Health Association, which promotes the interests of people with dementia and their carers and has local branches throughout the country. Providers of peer support use their lived experiences of overcoming distress to help others, in a relationship based on credibility, mutuality, hope and empowerment [17]. Prior to data collection, approval was obtained from the Norwegian Centre for Research Data (no. 56300).

### Data collection

In November 2018, the authors simultaneously conducted focus groups, each author with 9 of the 36 participants, lasting about 60 min. The focus group design allows participants to communicate, interact and share their experiences with each other [18] and provides participants with the opportunity to add further information to others’ statements. It also facilitates spontaneous and informal discussions about participants’ experiences and perceptions of the research phenomenon (i.e. participating in the PSP process). A lecture on the JLA approach and PSP process preceded the focus groups. During the focus groups, participants discussed the challenges they thought they would face if serving on a JLA steering group and during the PSP process. Participants were asked to reflect on the following topics guided by the authors: (1) the specific JLA method; (2) their preferred approach to participating in the process of identifying uncertainties; and (3) the most important factors for researchers to consider when recruiting and involving people with dementia and their carers in the PSP process.

### Data analysis

All interviews were audio-recorded and then transcribed verbatim. The transcripts were not returned to the participants for their comments; they were available only to the authors. The analysis, performed by all the authors, followed the thematic analysis procedures described by Braun and Clarke [19]. This six-step method is used to identify, analyse and report qualitative data patterns by (1) familiarizing oneself with the data, (2) generating initial codes, (3) searching for themes, (4) reviewing the identified themes, (5) defining and naming the themes and (6) preparing a report. We examined the ideas, conceptualizations and assumptions underlying the expressed content. Examples of the analysis process are shown in Table 1 with regard to the text units, codes, subthemes and the theme “Power of position, knowledge and language”. To preserve variability, encourage reflexivity and establish credibility, each author analysed the data independently. The themes and subthemes derived from the data were consolidated on the basis of researcher consensus.

**Table 1:** Summary of the thematic analysis related to the theme “Power of position, knowledge and language”

Text unit	Code	Sub-theme	Theme
If one contributes, one must be taken seriously. I gather that we are very popular with researchers because they will not receive funding if they don’t include service users. It’s rather scary. One doesn’t really know if research becomes more applicable with user involvement	Contribute Respect Researchers need Users Alibi Condition for funding Scepticism Applicable	Tension between different purposes	Power of position, knowledge and language
So, our participation in this, it’s to be able to help those coming after us. That’s the way it’ll be, so it becomes interesting to see if what we’ve contributed to the project can help others. That’s the way it has to be. Because we are past that stage, so we could just have turned our backs and not cared, because we are done with all of this, but that’s not who we are. I want to contribute. I want to make a difference to those coming after us	Focus on future Impact Altruism Responsibility Make a difference Contribution Participation	Involvement must have impact	
The research must have some consequences, must lead to something within health and social care or in politics, not least for the people in question. Research on causality might be too optimistic. As previously mentioned, it is my opinion that grasping the experiences of those with dementia and their carers is important for facilitation and obtaining insight	Consequence for health services Impact Consequence for politics Consequence for public users The importance of users’ experiences Realistic Relevance Facilitation Obtaining insight	Importance of knowledge for policy and health services	

To enhance trustworthiness and limit potential threats to validity, we used the four “trustworthiness” criteria described by Lincoln and Guba [20]: credibility, transferability, dependability and confirmability. Credibility was ensured through open-ended questioning, prolonged engagement with the data and articulation of a detailed description of methods. Transferability was achieved by providing an in-depth, detailed and descriptive analysis of the data and by quoting participants’ responses to substantiate the findings. To ensure dependability, the transcripts were reviewed several times and then checked and coded by each author. Interpretations were also based on consensus among the authors. Confirmability was achieved by substantiating each emergent theme with rich quotes extracted from the participants’ responses.

According to Berger [21], the position and reflexivity of the qualitative researcher is critical at all stages of the research process. Accordingly, the researchers’ professional background (i.e. healthcare personnel in the fields of physiotherapy, dietetics and nursing) and clinical experience (i.e. community healthcare) could affect the data collection and analytic procedures. The researchers are all females familiar with the language of the research context and able to address certain topics or pose follow-up questions in such a way that might positively influence (i.e. enrich) both the data quantity and quality. However, it is also possible that the researchers might overestimate between-participant similarities and consequently overlook individual differences in experience, which may impede the discovery and construction of new knowledge [22]. To avoid these problems in this study, the researchers maintained a constant sense of awareness during both the focus groups and data analysis to ensure that their preconceived notions did not affect the study findings. The coding strategy and the emergent subthemes, main themes and overarching theme are presented in Table 1.

## Results

There were 36 participants in the study. Participants’ characteristics can be found in Table 2.

**Table 2:** Characteristics of participants n (%) (*n* = 36)

**Gender**	
Female	28 (77.8)
Male	8 (22.2)
**Age**	
Under 30 years	0 (0)
30–39 years	1 (2.8)
40–49 years	2 (5.6)
50–59 years	3 (8.3)
60–69 years	17 (47.2)
70–79 years	12 (33.3)
80 years and over	1 (2.8)
**Education**	
Primary School	4 (11.1)
College	9 (25.0)
University college	13 (36.1)
University	9 (25.0)
**Number of years Peer Support**	
1–3 years	12 (33.3)
4–6 years	19 (52.8)
9 years or more	5 (13.9)

Thematic analysis yielded two main themes and seven subthemes, which were integrated into one overarching theme: “Creating empowering teams where all voices are heard” (see Table 3). The overarching theme incorporating the participants’ suggestions of the importance of equivalence in power, mutual agreement with and understanding of the goals, sufficient support, openness about each partner’s tasks and the bonds needed between the partners to sustain the enterprise, and expectations of positive outcomes.

**Table 3:** Overview of the steps in the analysing process

Overarching theme: Creating Empowering Teams Where All Voices Are heard	
Themes	Subthemes
Interaction of human factors, PSP process and the environment	• Diversity of carers • The person behind the disease • The person’s capability and features of the PSP • Experience-based research topics increase user relevance
Power of position, knowledge and language	• Tension between different purposes • Involvement must have an impact • Importance of knowledge for policy and health services

### Interaction of human factors, PSP process and the environment

The participants identified important prerequisites and precautions for participation in a PSP process by people with dementia and their carers. The participants emphasized that people with dementia and their carers are as diverse as any other societal group with different experiences and capabilities. Given this diversity and the diversity in the signs of dementia diseases, the participants identified barriers to and facilitators of PSP recruitment and participation by people with dementia and their carers (e.g. circumstances related to logistics).

#### Diversity of carers

The participants focused on their diversity, recognizing that carers differ as much as any other societal group in terms of age, resources, circumstances and family situation. Despite such differences, they also reported sharing many experiences. One participant said this:

“*Carers of persons with dementia are not a uniform group, so you’ll probably find as many different opinions there as you would in society in general.*”

The participants also spoke about the differences among them in terms of circumstances, resources, ages and family situations. Their unique carer roles depended on their relationship with those for whom they care (e.g. spouse vs. parent, younger vs. older adult). Since the carers also differed in age, they might be in different life stages:

*“You have young adults where the carer works full time, but if you get it at age 90, even though the other might also be old and frail in their own way, at least they don’t work. So even there, there’s a significant difference.”*

Furthermore, the participants clearly emphasized that it was important to participate in the PSP process to share experiences with others in the same situation. They saw participation as a way of helping to relieve bad feelings, such as shame, for other carers. One participant said this:

*“Very many people I talk to say, they have felt that pain. I recognize it. I’m like you – I know something about being on the verge of shame. I’ve tasted the feeling of life being meaningless. Not to mention empty.’ I’m afraid of being exposed as a useless carer – I’ve felt like a total failure. Although I now feel confident. To share one’s innermost thoughts with others, your most shameful thoughts – I think that is of value to others.”*

The participants also said that it was common to experience joyful moments and share pleasant experiences with the person affected with dementia:

*“I want to say that all those years with Alzheimer’s, it was not only sorrow. On the contrary, there was a lot of happiness, many bright moments and much good. And I had my husband home until the end. I was younger, so I was able to.”*

#### The person behind the disease

The participants emphasized that no one should assume that dementia automatically means confusion, an inability to express oneself or inability to participate in a team-based research discussion. Participants noted the importance of seeing the person behind the disease:

*“See the person, not the disease, see the person. That, at least, is important.”*

People with dementia have life histories from before their disease that may enable them to participate in research and the PSP process despite the disease. Most of them would have been active, working and participating in various societal activities:

*“Yes, they are resourceful people, well-educated people who have experienced stuff earlier in life. You could say they are intelligent people.”*

Facilitating participation in the PSP process by those with dementia and including their experiences may help to uncover research areas that have received inadequate attention. One participant pointed out the importance of sufferers’ voices being heard in research:

*“Previously we, as the carers, have been their spokespeople, right, but it’s not a given. If they’ve lost the ability to speak in an early phase, it’s not certain that we assume correctly, and if they don’t lose the ability to speak, it’s not really certain that we think the same way they’re thinking. So, if they’re allowed to express themselves without interference, it’s definitely much better, as long as they can.”*

Paying extra attention to those with frontal dementia, who may have impaired perceptual self-knowledge, is needed to facilitate participation. Such individuals are often younger and represent an important group. One participant said this:

*“Some get it at a young age – the person with dementia is young when they lose introspection – and it’s not so easy to participate then. And that group should also be represented.”*

#### The person’s capability and features of the PSP process

In order for persons with dementia and their carers to be able to participate in the PSP process, support must be provided if necessary for the carers. The participants were concerned that the carers and those with dementia should receive the information about the project needed for participation in the PSP process. They said that their involvement in the PSP process would be demanding in terms of the time and energy needed:

*“Of course, it can be taxing to participate in the whole process, which seems fairly long, and it isn’t easy to commit to participating in a process like this, that might last more than half a year, especially not in the period when you are most burdened.”*

Noting that carers needed energy to participate in a long PSP process, they said that facilitation had to be optimal for those with dementia and their carers. Some carers cannot leave the house without the person with dementia:

*“And you must facilitate participation. That was a big problem, I could barely leave my husband for long enough to get to the mailbox.”*

Facilitation includes helping the participants to understand the project and clarifying the researchers’ expectations of the partnership with the participants. One participant commented thus:

*“[It is necessary] to set aside time and resources for collaboration in the planning phase, and to clarify expectations as to how the service user should contribute. I imagine it could become difficult when it comes to interpreting the information gathered.”*

Continuously participation in all PSP steps was highlighted by several participants, who emphasized that it could be difficult for people with dementia to participate in the entire PSP process. One participant said this:

*“But carers should, if possible, contribute at all stages of the research process if they are willing. Naturally, challenges must be discussed openly all along. The person with dementia might not have the necessary resources to do it. I would think that the carers represent continuity [to the people with dementia]. They have the experience alongside the service users.”*

The participants also emphasized the importance of carers being with the person with dementia throughout the PSP process because the disease would progress during the process. One participant said the following:

*“I think you’ll get more service users in the early stages of the disease, before it’s very far gone, but that makes it important to include the carers all along, because if you are to follow a research process through all the stages, it will take time and the disease progresses all the time. The carer then provides the continuity [to the people with dementia], having the experiences alongside the service user. So, I think this – but I also think there will be some barriers when it comes to recruitment – because they have to have acknowledged at an early stage the fact that they have dementia.”*

The focus group participants suggested that to enable people with dementia to participate in a steering group, all steering group members should speak slowly and clearly:

*“Be clear. Don’t speak quickly. Many things are details, but they are important. Use short sentences. Even at an early stage, they have difficulties with long sentences and large volumes of information, but they have much to contribute if only we do it the right way.”*

The focus group participants also said that PSP participants should be recruited through organizations, so that potential participants understand that this is a serious process. One participant said this:

*“I think it might be advantageous to recruit service users through an organization if you want the service user to be able to convey the needs of the group represented by the organization.”*

#### Experience-based research topics increase user relevance

Mastering everyday life is important to carers and those for whom they care. The participants explained that understanding what it is like to live with dementia makes it easier to master their carer role. The focus group participants wanted research to focus more on their experiences as carers.

They reported that being a carer and peer supporter means that they experience several areas about which they need more knowledge. They also want research to contribute to societal understanding of what it means to be affected by dementia.

*“And understanding. Yes, research into how to create understanding. That others understand those who are affected with dementia – not just carers. Little understanding around. Family, friends, workplace, everywhere. Researching how understanding can be achieved would be interesting and important. I think that a society with appreciation for weakness would be a better society for most.”*

The participants said that they wanted research to focus on optimizing everyday life for both the people with dementia and their carers. They also wanted improved knowledge for the most vulnerable carers, children and young people, about how it is to care for a parent with dementia:

*“We talked about being a child, a youth with parents with dementia and I said that – Has any research been done into how they fare later in life, when they’ve grown up with, perhaps over a long period, a mother or a father with dementia? And I’m personally very concerned about this, put it that way, but I feel it would be interesting on a more general basis too.”*

### Power of position, knowledge and language

The participants observed a disparity in power of knowledge and language across the different positions of researcher, health professional and patient. The researcher needs to speak using language that the participants can understand and must feel that the participants’ roles in the PSP process are important. The participants noted that researchers often approach clinical practice with ready-made solutions and definitive answers, which may lead to tension between differing intentions. It is important for the participants not to feel that they are token participants in the process, an alibi for researchers to obtain funding, but rather that their experiences are important and that their involvement has an impact.

#### Tension between different purposes

Participants in the four focus groups highlighted the need for awareness that tension between participants’ different purposes could influence carers’ motivations for engagement in the PSP process. Both carers and the people with dementia must be taken seriously and must experience a sense of having been heard. One participant commented thus:

*“If one contributes, one must be taken seriously. I gather that we are very popular with researchers because they will not receive funding if they don’t include service users. It’s rather scary. One doesn’t really know if research becomes more applicable with user involvement.”*

The participants explained that carers have years of experience, which makes them the “experts” on their situation, as one participant said:

*“It’s what I know, and I feel that I have something to contribute for others in the same situation that I’ve been in for 25 years. I perceive that my experience is important. I also feel that others’ experiences are beneficial to me.”*

To involve people with dementia and their carers, researchers must understand these user groups and how to interact with them. One participant commented thus:

*“We also know that researchers must behave in a down-to-earth manner, to gain people’s trust.”*

These informants do not want simply to be the researcher’s funding alibi. They need to feel equal to the researcher, to be an important part of the research:

*“It should not just be an alibi in order to be granted research funding.”*

#### Involvement must have an impact

The participants have ideas about how research can enrich and benefit its users. They know that today’s research may not benefit them or their situation, but they want to help make improvements for others in the future who are in the same situation:

*“So, our participation in this, it’s to be able to help those coming after us. That’s the way it’ll be, so it becomes interesting to see if what we’ve contributed to the project can help others. That’s the way it has to be. Because we are past that stage, we could have just turned our backs and not cared, because we are done with all of this, but that’s not who we are. I want to contribute. I want to make a difference to those coming after us.”*

The participants wanted to have a real impact on research through genuine user involvement. Gaining knowledge and information about the PSP process was at the core of the participants’ feedback:

*“There must be a purpose to our participation, let’s put it that way. We must have a say in prioritizing.”*

The informants were divided as to whether it was enough to know that research might benefit people in a similar situation in the future or if it was necessary to experience benefits in the present. As one participant said it:

*“There must be something in it for me as a service user too, sort of. Is this useful to me and my problems, to get answers?”*

Another said the following:

*“A research project cannot help me anymore. My husband is now dead, he passed away last month, so it can’t … so I’ve seen this through to the end, so it’s what can help others coming after, that’s my motivation for being a peer too. It’s so that it can be of use to others. That’s the future.”*

From the participants’ perspective, because the PSP process is demanding, they needed to understand their role and purpose in the process. For them, before joining a steering group, it was important that they understood what was expected of them in the PSP process:

*“Clear outlines. First of all, be clear on the purpose of user involvement in the project in question. What is the reason, what is the goal, in what phases is it most important and to what extent can they be involved? Project management must be clear about the service users’ role and clarify expectations as to the use of time, type of contribution, co-authoring, remuneration, etc.”*

Information and follow-up at the end of the project are also important to the participants. As one participant said, they wanted to know whether their contribution was used and what came out of the project:

*“Often you hear nothing afterwards. Complete silence. The information you’ve given is just stored in a database or something. Involvement and follow-up are very important. One must know the what, how and why.”*

#### Importance of knowledge for policy and health services

The carers perceived that health care users’ experiences of illness can make important contributions to clinical research–based knowledge. Including people with dementia, carers and clinicians in research should help to improve everyone’s perspective on the experience of living with dementia. Through their contribution to the PSP process, the participants wanted those in policy and health services to hear their voices. They noted that research must have consequences for both people with dementia and their carers; these consequences include policy changes in the health services:

*“The research must have some consequences, be able to lead to something within health and social care or in politics, not least for the people in question. Research on causality might be too optimistic. As previously mentioned, it is my opinion that grasping the experiences of those with dementia and their carers is important for facilitation and obtaining insight.”*

The participants noted the importance of meeting dedicated and competent clinicians within the healthcare services. They also considered grant money to be a political issue, as one participant commented:

*“The politicians say they’re concerned with dementia care – but they do not put any money into it – and do not put research results into use.”*

Carers want policymakers to have a better understanding of their situation and they want to be involved in political decisions about dementia care:

*“Take this small example. These days there’s a lot of talk about dementia-friendly communities, and then they talk about how the person is going to find their way back. But really, that’s not an issue I recognize at all. It’s something politicians act upon to make themselves look better and show that they care about dementia in a tiny, very visible area. That annoys me.”*

The participants need to feel that they are influencing research and to see changes in the health care system:

*“So, everything I had been saying for five hours, it didn’t even penetrate the skin. I don’t understand the politicians who are supposed to grant us money.”*

## Discussion

To our knowledge, this study is the first to investigate carers’ reflections on how they and people with dementia may participate in a PSP process. The overall results highlight the importance of both people with dementia and their carers participating in PSP processes. The participants emphasized how important it is that research topics should have a positive impact on their everyday life, policy development and healthcare services. The carers asked for transparency, disclosure, open communication and convenience to ensure that engagement is easy, feasible and flexible. A previous study has noted that those who experience illness and medical care directly, such as patients, family or carers, are uniquely positioned to contribute to research efforts to understand and improve diagnosis, management, treatment and healthcare delivery [23]. Moreover, patients and members of the public possess experimental knowledge that researchers may lack [24]. Our findings support this.

A common element that emerged from our findings is that involving people with dementia requires the researchers to see the person behind the disease and to facilitate their participation in meetings. This is consistent with findings from previous studies, one of which pointed out that people with dementia are among the most vulnerable members of society, with needs over and above those without cognitive impairment, and consequently they need additional support [25]. Another study noted that those responsible for the research process must have relevant communication skills and knowledge about dementia to empower people with dementia and to ensure they have positive experiences by participating in a PSP [26]. Such findings highlight the importance of attitudes, towards both people with dementia and people with dementia who contribute to research [27]. It is also important to include successful, fun moments, despite the dementia [26].

Another specific recommendation for involving seriously ill people in the research process is to consider their special care needs in order to ensure sensitive facilitation, because the research process can be upsetting for participants [28]. Our participants noted the importance of speaking slowly and in short sentences during meetings attended by people with dementia.

If the person with dementia is unable to participate in research because of their disease type or severity, carers may need support to enable their own participation (e.g. help caring for the person with dementia, so they can be away from home). This finding is in line with that found elsewhere that challenges for patients and carers taking part in projects are many, mainly in terms of the practicalities of being able to attend when a patient is not well enough [29]. Furthermore, as a group, carers to people with dementia are more vulnerable than others in their age group and experience higher rates of stress [5], which may be a barrier to participating in the PSP process.

In terms of PPI and user involvement, the participants highlighted the importance of being taken seriously by the researchers. This finding is consistent with the previous finding that meaningful patient engagement evolves from authentic partnership [30]. All parties should consider the capacity, skills and interests of both the patients and researchers and use these to establish mutually beneficial roles. Our participants’ statements were consistent with the observation made elsewhere that meetings can be structured so that discussions occur within small, facilitated groups in which many find it easier to contribute [31].

Our informants stated that user involvement in research presupposes that the experiences of patients and their carers are meaningful. Expectations should be expressed clearly and concisely. Confidentiality and trust are also crucial, especially when a patient or carer shares sensitive, personal or emotional information. All involved must know that they can stop the discussion if they feel uncomfortable or distressed [29].

The participants in this study clearly do not want to be perceived as an alibi for researchers seeking funding. Previous research has suggested that closing the gap between researchers and research users is improved by using PPI to set the research agenda [32]. However, our findings indicate that researchers need to look more closely at the concept of power. A central point of departure in power theories is Max Weber’s classic definition of power as “the ability of an individual or group to achieve their own goals or aims when others are trying to prevent them from realizing them” [33]. In the research literature, such power is referred to as “power over” and is linked to domination and coercion, or a process with a loser and a winner. Thus, when someone has more power, others have less [34]. For example, experts may ignore the goals of their patients as a result of the belief that they know what is best for the patients. However, we must also recognize structural power (i.e. mechanisms within organizational and physical structures). Such power may be more difficult to see but it can affect how patients feel by, for example, creating unfavourable environments [35]. The literature also describes discursive power, or the power to define the understanding of a phenomenon. A discourse is a set of opinions, metaphors, representations, images, stories and statements that produce a specific version of events [35]. If a discourse becomes dominant and is internalized as an invisible truth, it becomes hegemonic power [36]. As our findings show, this power can be seen in the context of language. The participants emphasized the importance of researchers using language that they could understand and said that they need to feel that they are on the same level as the researcher. Using language that everyone involved understands may represent a partial shift of power from the researchers to the participants. Using a common language may also help researchers to stay connected to the “real world” to which their research should apply.

PPI can been see as an empowerment-associated approach, focusing on “power with”, “power to” and “power from within” rather than “power over” [34]. “Power with” refers to the capacity to achieve goals together, rather than at another’s expense; affective bonds are strengthened through this positive process by creating a proliferation of power [37]. “Power to” is “generative or productive power that creates new possibilities and actions without domination” [38] and “power from within” is associated with a belief in one’s inner strength and self-empowerment [34]. Our results reflect these constructs insofar as the participants feel self-empowered, as shown by such comments as *“it should not just be an alibi in order to be granted research funding”,* expressed in relation to the need to feel authentic user involvement and avoid a negative “power over” approach. Previous research also indicates that patient engagement improves research findings and that new information empowers patient advocates [30]. The same study suggests that authentic collaborations between patients, healthcare professionals and researchers are essential to the success of research and, in turn, to its applicability to improving health outcomes [30]. Similarly, the current study found that meaningful engagement is necessary in such collaborations, helping to identify value-added roles and to assure participants that the research has an impact, including helping politicians to understand the importance of funding to improve healthcare services.

### Limitations

As in all qualitative research, our findings are text-bound to the participants and study setting [39], in our case a Norwegian-specific context. A strength is that the volunteers represented different geographical regions, yielding a wider range of perspectives on the important determinants for participating in the PSP process. However, their views may not represent the broader population of carers and people with dementia. The value of our findings is based on the expanded discussions in four focus groups. The data produced were very rich, and hopefully we reached data saturation [40], in line with idea of “information power” [41], although further interviews with the carers might have strengthened our data and even led to other findings and conclusions.

### Implications for researchers and policymakers

Clinicians and policymakers should be more aware of the challenges to JLA participating in a PSP process faced by carers, who may experience this as an emotional burden. Nevertheless, there is strong potential for the research community to support research participation by people with dementia and their carers.

## Conclusion

Carers suggested that both they and the people with dementia for whom they care are able to contribute to the PSP process when given sufficient support. The involvement of these groups is important for setting healthcare research agendas, developing research projects that increase awareness and knowledge about their circumstances, and improving researchers’ and policymakers’ understanding of and insight into their unique situations. Their involvement helps in the identification of relevant research topics and improves relevance of research outcomes to users. Successful participation requires emphasis on equity in power issues, open dialogue and the importance of users’ voices in the decisions that affect them.

## Supplementary information

**Additional file 1.** Topics for the focus groups

## Data Availability

The datasets generated and analysed during the current study are not publicly available due to the need to protect participant confidentiality but are available from the corresponding author on reasonable request.

## Abbreviations

JLA: James Lind Alliance
PPI: Patient and public involvement
PSP: Priority Setting Partnership
