# Supplementary material for: It takes two to tango: carers’ reflections on their participation and the participation of people with dementia in the James Lind Alliance process
Source: BMC Geriatr. 2020 May 14;20:175. doi: 10.1186/s12877-020-01570-3 (PMC7227228; doi:10.1186/s12877-020-01570-3)
Supplement: Supplementary file 1 — Additional file 1. Topics for the focus groups [file 12877_2020_1570_MOESM1_ESM.docx]

**Topics for the focus groups**

- What are your reflections and thoughts about involving people with dementia and their carers in a JLA PSP process?
- Reflect on barriers and facilitators for involving people with dementia and their carers in a JLA PSP process
- Discuss what you think is of importance if we as researchers want to involve carers of people with dementia in research?
- What other factors in terms of user participation do you think are important in research related to people with dementia?
- Any other things you would like to share about user involvement in research?
